# Association of Kidney Function with Infections by Multidrug-Resistant Organisms: An Electronic Medical Record Analysis

**DOI:** 10.1038/s41598-018-31612-1

**Published:** 2018-09-06

**Authors:** Guobin Su, Hong Xu, Emilia Riggi, Zhiren He, Liming Lu, Bengt Lindholm, Gaetano Marrone, Zehuai Wen, Xusheng Liu, David W. Johnson, Juan-Jesus Carrero, Cecilia Stålsby Lundborg

**Affiliations:** 1 Global Health – Health Systems and Policy, Department of Public Health Sciences, Karolinska Institutet, Stockholm, Sweden; 2 Department of Nephrology, Guangdong Provincial Hospital of Chinese Medicine, The Second Affiliated Hospital, Guangzhou University of Chinese Medicine, Guangzhou city, Guangdong Province China; 3 Department of Medical Epidemiology and Biostatistics, Karolinska Institutet, Stockolm, Sweden; 4 Division of Renal Medicine and Baxter Novum, Department of Clinical Science, Intervention and Technology, Karolinska Institutet, Stockholm, Sweden; 5 Department of Brain and Behavioral Sciences, Unit of Medical Statics and Genomics, University of Pavia, Pavia, Italy; 6 Key Unit of Methodology in Clinical Research (KUMCR), Guangdong Provincial Hospital of Chinese Medicine, The Second Affiliated Hospital, Guangzhou University of Chinese Medicine, Guangzhou city, Guangdong Province China; 7 Department of Nephrology, Princess Alexandra Hospital, Brisbane, Australia; 8 Centre for Kidney Disease Research, University of Queensland, Brisbane, Australia; 9 Translational Research Institute, Brisbane, Australia

## Abstract

Antibiotic resistance is a major global health threat. High prevalences of colonization and infection with multi-drug resistance organisms (MDROs) have been reported in patients undergoing dialysis. It is unknown if this finding extends to patients with mild and moderate/severe kidney disease. An observational study included all adult incident patients hospitalized with a discharge diagnosis of infection in four hospitals from Guangzhou, China. Inclusion criteria: Serum creatinine measurement at admission together with microbial culture confirmed infections. Exclusion criterion: Undergoing renal replacement therapy. Four categories of Chronic Kidney Disease Epidemiology Collaboration (CKD-EPI) estimated glomerular filtration rate (eGFR) were compared: eGFR ≥ 105, 60–104 (reference), 30–59, and <30 ml/min/1.73 m^2^. The odds ratio of MDROs, defined as specific pathogens (*Staphylococcus aureu*s, *Enterococcus spp*., *Enterobacteriaceae*, *Pseudomonas aeruginosa* and *Acinetobacter spp*.) resistant to three or more antibiotic classes, were calculated using a multivariable logistic regression model across eGFR strata. Of 94,445 total microbial culture records, 7,288 first positive cultures matched to infection diagnosis were selected. Among them, 5,028 (68.9%) were potential MDROs. The odds of infections by MDROs was 19% and 41% higher in those with eGFR between 30–59 ml/min/1.73 m^2^ (Adjusted odds ratio, AOR): 1.19, 95% CI:1.02–1.38, P = 0.022) and eGFR < 30 ml/min/1.73 m^2^ (AOR: 1.41, 95% CI:1.12–1.78, P = 0.004), respectively. Patients with impaired renal function have a higher risk of infections by MDROs. Kidney dysfunction at admission may be an indicator for need of closer attention to microbial culture results requiring subsequent change of antibiotics.

## Introduction

Antibiotic resistance (ABR) is a major growing global threat because even common infections and minor injuries may becoming life threatening due to the increasing incidence of infections caused by multi-drug resistant organisms (MDROs)^1^. As recently stated by the United Nations (UN) and World Health Organization (WHO), ABR has escalated into a global crisis that must be prioritized^2,3^.

Chronic kidney disease (CKD) as well is a global health problem, affecting 10–15% of the population worldwide^4,5^. Patients with CKD are not only the victims of infection, but plausibly also a reservoir of antibiotic-resistant pathogens. An increased risk of infection has been reported in individuals with CKD, especially in those undergoing dialysis^6^. Higher rates of infection in patients with CKD result in more frequent use of antibiotics^7^ and more frequent hospitalization^6,8,9^, thereby increasing their exposure to microbes, including MDROs^10^.

High prevalences of colonization by, and infection due to MDROs, such as *vancomycin-resistant enterococci* (VRE), or *methicillin-resistant Staphylococcus aureus* (MRSA), have been reported in patients undergoing dialysis^11–14^. However, it is currently unknown if non-dialysis CKD patients are at higher risk of infections caused by MDROs. Studying these infections is relevant given the potential nephrotoxicity of second and third line antibiotics^15^. What is worse, these patients can potentially transmit MDROs to patients in other sections of the healthcare facilities.

Since the recognition of CKD remains low^4^, pre-admission baseline kidney function, such as estimated glomerular filtration rate (eGFR), is not always available when patients are admitted to hospital. In clinical practice, eGFR at the time of admission represents an important indicator of kidney function that may help to inform clinical decision making, even though reduced eGFR at admission may represent acute and/or chronic kidney dysfunction.

The aim of this study was to explore the association between kidney function at admission and the risk of infections by MDROs in patients hospitalized with infections. Secondly, we tried to identify specific microbial patterns of infection among them. The existence of such patterns would help to inform public planning strategies to cope with antibiotic resistance.

## Results

### The flowchart of included microbial cultures

**Figure 1:**
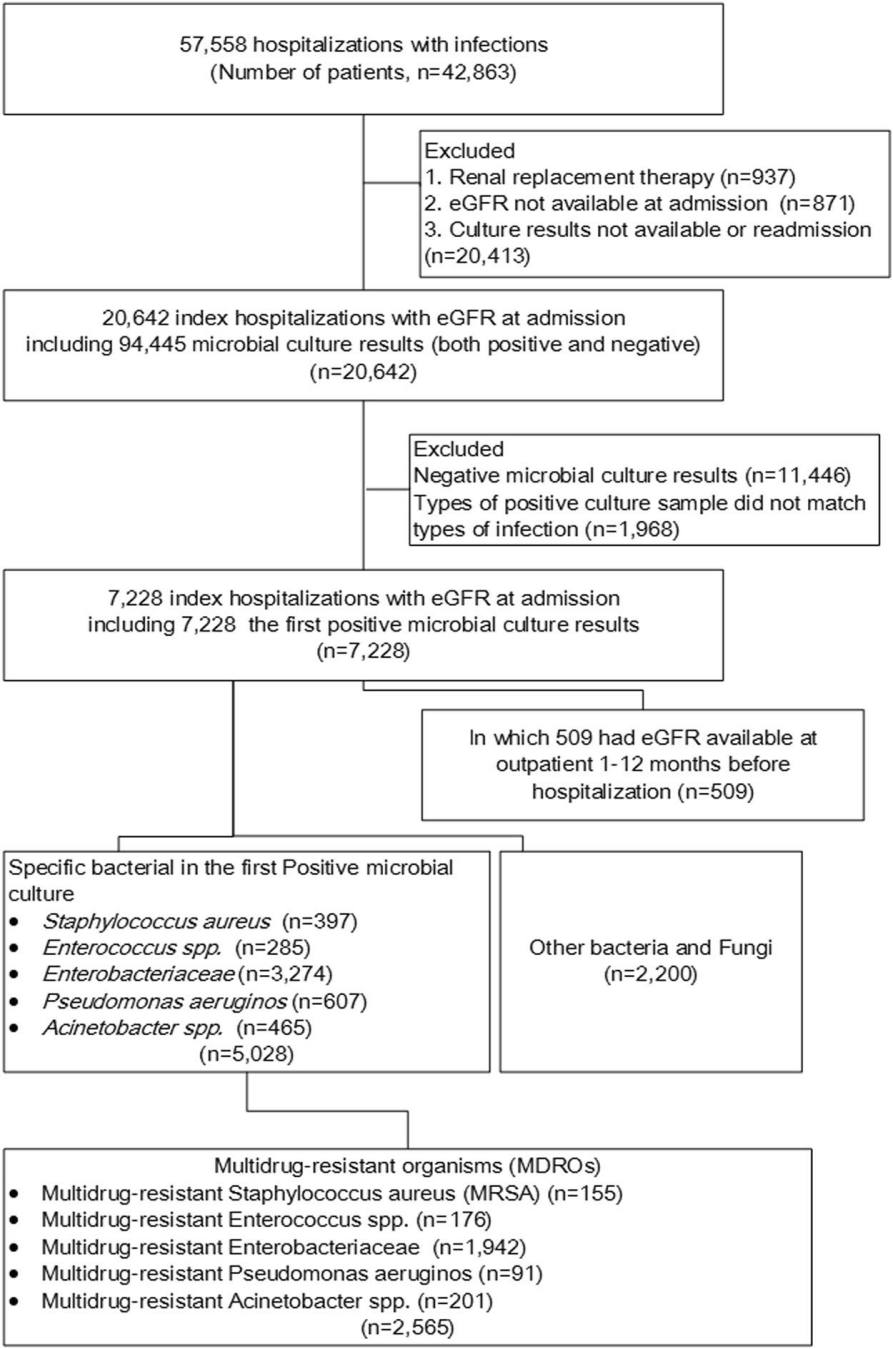
Study Flowchart.

In total, 42,863 patients had 57,558 hospitalizations with infections during the study period. In their first hospitalization, 20,642 patients had 94,445 microbial culture results (both positive and negative). After excluding those without positive microbial results or infection diagnoses that were not matched to a culture confirmed sample, a total of 7,228 incident hospitalized patients with the first positive microbial culture results were included. Of these, 509 patients had eGFR available at a prior outpatient visit 1–12 months before hospitalization. Those samples with positivity for *Staphylococcus aureu*s, *Enterococcus spp*., *Enterobacteriaceae*, *Pseudomonas aeruginosa* and *Acinetobacter spp*. accounted for 68.9% (5,028/7,228) of the first positive cultures. Among them, 51.0% (2,565/5,028) were MDROs (Fig. 1).

### Baseline demographics of patients in different groups

Of included patients (n = 5,028), 58.6% were females and the mean age was 70 years. The majority (n = 3,038, 60.4%) had normal or near-normal kidney function (eGFR of 60–104 ml/min/1.73 m^2^). The most common comorbidities were cerebrovascular disease (35.0%), diabetes mellitus (30.3%), chronic obstructive pulmonary disease (18.6%), and cancer (17.9%). Undefined infection-related hospitalizations (IRHs) were the most common IRHs (64.0%), followed by community-acquired (26.0%) and healthcare-associated IRHs (10.0%). The prevalence of comorbidities was higher in lower eGFR categories (Table 1).

**Table 1:** Baseline demographics of patients with first culture positive bacteria** stratified by eGFR categories.

eGFR at admission	eGFR at admission (ml/min/1.73 m^2^)					
	Total	≥105	60–104	30–59	<30	^*^P value
	(n = 5,028)	(n = 689)	(n = 3,038)	(n = 968)	(n = 333)	
Age (years, median) [interquartile range]	70 [58–79]	46 [36–56]	71 [60–79]	78 [70–83]	76 [65–81]	<0.001
Female, n (%)	2,948 (58.6)	393 (57.0)	1,772 (58.3)	564 (58.3)	219 (65.8)	0.05
Charlson comorbidity index	1 [0–2]	1 [0–2]	1 [1–2]	2 [1–3]	2 [1–3]	<0.001
*Single Comorbidities*						<0.001
Myocardial infarction, n (%)	90 (1.8)	4 (0.6)	42 (1.4)	32 (3.3)	12 (3.6)	<0.001
Congestive heart failure, n (%)	344 (6.8)	8 (1.2)	150 (4.9)	128 (13.2)	58 (17.4)	<0.001
Peripheral vascular disease, n (%)	57 (1.1)	2 (0.3)	26 (0.9)	21 (2.2)	8 (2.4)	<0.001
Cerebral vascular disease, n (%)	1,783 (35.0)	128 (18.6)	1,082 (35.6)	449 (46.4)	124 (37.2)	<0.001
Dementia, n (%)	61 (1.2)	4 (0.6)	44 (1.5)	9 (0.9)	4 (1.2)	0.2
Chronic obstructed pulmonary disease, n (%)	933 (18.6)	88 (12.8)	606 (20.0)	190 (19.6)	49 (14.7)	<0.001
Connective tissue disease, n (%)	105 (2.1)	21 (3.1)	59 (1.9)	19 (2.0)	6 (1.8)	0.34
Peptic ulcer, n (%)	158 (3.0)	14 (2.0)	88 (2.9)	38 (3.9)	18 (5.4)	0.01
Paraplegia, n (%)	10 (0.2)	7 (1.0)	3 (0.1)	0 (0.0)	0 (0.0)	<0.001
Diabetes, n (%)	1,523 (30.3)	107 (15.5)	892 (29.4)	371 (38.3)	152 (46.0)	<0.001
Cancer, n (%)	900 (17.9)	170 (24.7)	558 (18.4)	144 (14.9)	28 (8.4)	<0.001
Severe liver disease, n (%)	49 (1.0)	6 (0.9)	33 (1.1)	8 (0.8)	2 (0.6)	0.76
Types of infection-related hospitalizations						<0.001
Total, n (%)	5,028 (100.0)	689 (100.0)	3,038 (100.0)	968 (100.0)	333 (100.0)	
Community-acquired IRHs, n (%)	1,309 (26.0)	216 (31.4)	773 (25.4)	242 (25.0)	78 (23.4)	0.006
Hospital-acquired IRHs, n (%)	504 (10.0)	106 (15.4)	295 (9.7)	84 (8.7)	19 (5.7)	<0.001
Undefined IRHs, n (%)	3,215 (64.0)	367 (53.3)	1,970 (64.9)	642 (66.3)	236 (70.9)	<0.001

^*^Mann-Whitney U test or Analysis of variance or Chi-square test or rank-sum test.

**Staphylococcus aureus, Enterococcus spp., Enterobacteriaceae, Pseudomonas aeruginos, Acinetobacter spp.

Data from four hospitals of Guangdong Provincial Hospital of Chinese Medicine, Guangzhou, China.

### Culture positive rates

In the first hospitalization, 20,642 patients had 94,445 microbial culture results with eGFR available at admission. The culture-positive rate was 21.8%, 20.8%, 30.3%, 10%, 25% in overall, sputum, midstream, venous blood and other types of specimen, respectively. A higher overall culture-positive rate was observed as eGFR declined (P = 0.06). When categorized according to specific sample source, the same pattern remained only in the sputum (P < 0.01). There were no significant differences observed for culture positive rates in midstream urine (P = 0.6), venous blood (P = 0.7) or other specimen types (P = 0.7) (Supplementary Table 3).

### Infection and microbial pattern in the first positive culture

Respiratory tract, genitourinary and bloodstream infections were the leading infections in these patients. (Supplementary Fig. 1) In 7,228 first positive microbial cultures, Gram-negative (G−) bacteria were the most commonly detected bacteria in the positive cultures, followed by Gram-positive (G+) bacteria, mycoplasmataceae and fungi. Compared to patients with eGFR 60–104 ml/min/1.73 m^2^, the proportion of G− bacteria decreased (from 68% to 63%) across decreasing eGFR strata, while the proportion of G+ increased (from 14% to 18%). Among the G+ bacteria noted, *Staphylococcus aureus (SA)* was the most common among patients with eGFR ≥ 30 ml/min/1.73 m^2^, and *Enterococcus spp*. the most common one among patients with eGFR < 30 ml/min/1.73 m^2^. Among the G− bacteria, *Escherichia coli (E.coli)* was the most common of all, followed by *Klebsiella* (Supplementary Fig. 2).

### MDROs in the first positive culture

**Figure 2:**
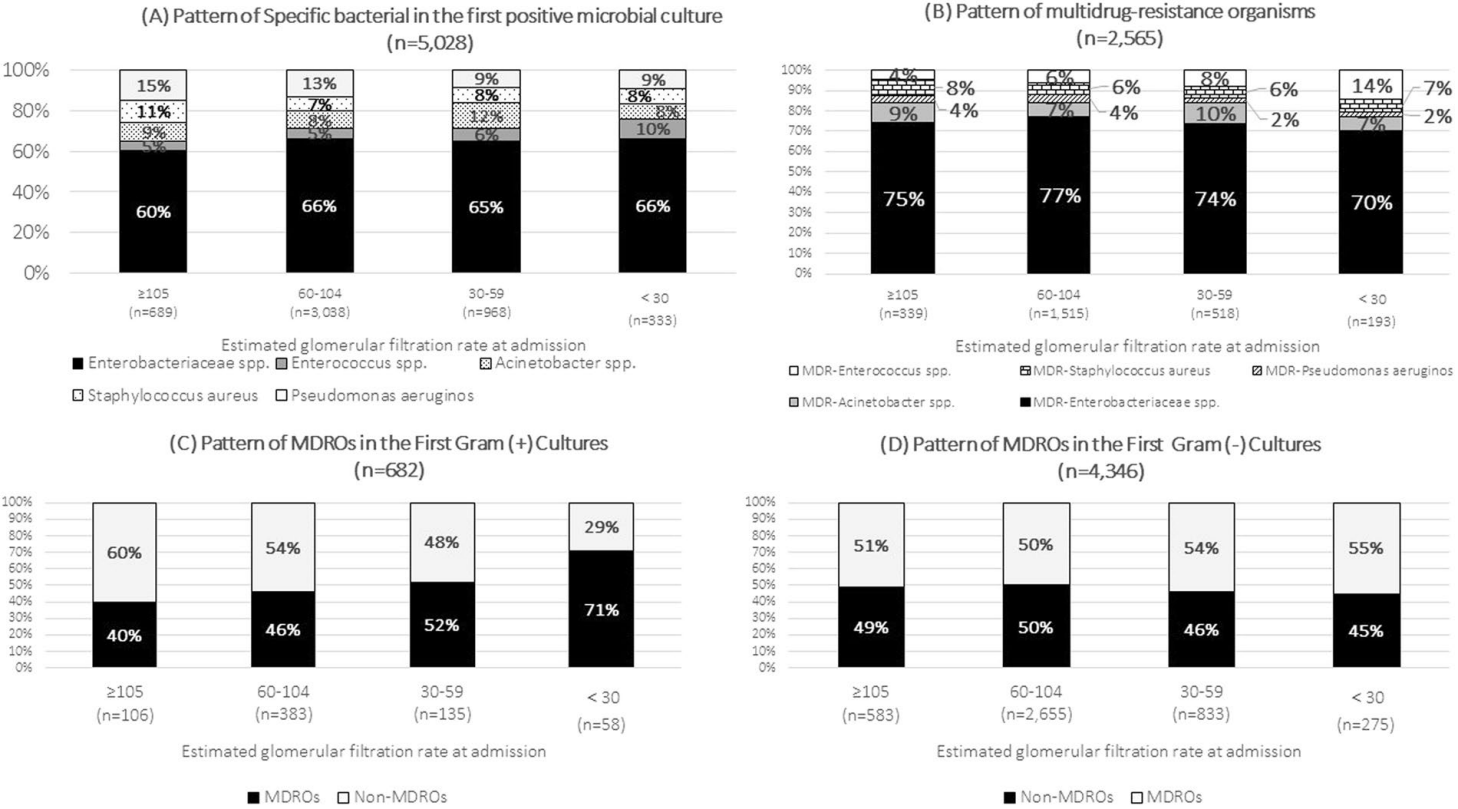
The pattern of multidrug-resistance organisms in first positive microbial culture pattern by eGFR categories: (**A**) Pattern of Specific bacterial in the first positive microbial culture; (**B**) Pattern of multidrug-resistance organisms; (**C**) Pattern of MDROs in the First Gram (+) Cultures; (**D**) Pattern of MDROs in the First Gram (−) Cultures.

In 2,565 MDROs, a different pattern in the first positive culture was observed according to different eGFR categories. G+ MDROs increased while G− MDROs decreased from eGFR between 60–104 ml/min/1.73 m^2^. *Enterobacteriaceae spp*. were the predominant MDROs, followed by *Enterococcus spp*., *Acinetobacter spp*., SA and *Pseudomonas aeruginosa*. Compared with eGFR between 60–104 ml/min/1.73 m^2^, the proportion of *Enterococcus spp*. started to rise as the eGFR declined. (Fig. 2).

The crude MDRO proportions in the first positive cultures were 49.2%, 49.9%, 53.5% and 58.0% in patients with eGFR ≥ 105, 60–104, 30–59 and <30 ml/min/1.73 m^2^, respectively. Regarding specific pathogen subgroups, the crude multidrug-resistant ratio increased with decreasing eGFR for MRSA, *Enterobacteriaceae spp., Enterococcus spp*. and *Acinetobacter spp*., but decreased for *Pseudomonas aeruginosa*. The same trend was observed in the stratified analyses by disease subgroups and specimen subgroups, but not in venous blood specimen subgroups (Table 2).

**Table 2:** MDROs in the first positive culture result in different groups by eGFR at admission categories.

The first culture positive was MDRO*	eGFR at admission (ml/min/1.73 m^2^)						
	MDRO/Total	≥105	60–104	30–59	<30	P value*	P trend**
Overall MDROs***, **n** (**%**)	2,565/5,028	339 (49.2)	1,515 (49.9)	518 (53.5)	193 (58.0)	0.01	0.003
**Pathogen**							
Multidrug-resistant *Staphylococcus aureus*, n (%)	155/397	27 (36.5)	86 (38.2)	29 (39.7)	12 (52.0)	0.56	0.31
Multidrug-resistant *Enterobacteriaceae*, n (%)	1,942/3,274	252 (60.6)	1,172 (58.4)	383 (60.8)	135 (61.4)	0.57	0.2
Multidrug-resistant *Pseudomonas aeruginosa*, n (%)	91/607	13 (12.8)	62 (15.8)	12 (14.5)	4 (13.3)	0.87	0.56
Multidrug-resistant *Enterococcus spp*., n (%)	176/285	15 (46.9)	92 (58.2)	41 (66.1)	28 (84.9)	<0.01	<0.01
Multidrug-resistant *Acinetobacter spp*., n (%)	201/465	32 (49.2)	103 (40.4)	53 (44.2)	13 (52.0)	0.46	0.26
**Disease**							
Single pneumonia with sputum specimen, n (%)	487/1,169	74 (41.8)	291 (40.9)	87 (41.0)	35 (50.0)	0.53	0.04
Single UTIs with urine specimen, n (%)	1,284/2,151	144 (63.2)	759 (58.3)	271 (60.6)	110 (63.2)	0.35	0.08
All sepsis with positive microbial culture, n (%)	482/893	70 (50.7)	232 (52.1)	120 (56.3)	60 (61.9)	0.25	<0.001
**Specimen**							
Sputum, n (%)	630/1,678	87 (35.4)	378 (36.3)	127 (41.2)	38 (45.8)	0.15	0.04
Midstream urine, n (%)	1,397/2,307	152 (62.8)	831 (59.3)	299 (62.0)	115 (63.5)	0.46	0.14
Venous blood, n (%)	96/201	12 (33.3)	62 (54.6)	16 (44.4)	3 (30.0)	0.08	0.05

^*^Chi^2^ test in all subgroups; **The nonparametric test for trend across ordered groups from group 60–104 to group <30.

**MDROs: multi-drug resistance organism; Only applied in the five types of bacteria: *Staphylococcus aureus, Enterococcus spp., Enterobacteriaceae, Pseudomonas aeruginos, Acinetobacter spp*.; Resistant to three or more antimicrobial classes;

eGFR: estimated glomerular filtration rate. UTI: urinary tract infection.

Data from four hospitals of Guangdong Provincial Hospital of Chinese Medicine, Guangzhou, China.

The results of multivariable logistic regression models showed increased odds of MDROs across lower eGFR strata. Compared to eGFR 60–104 ml/min/1.73 m^2^, the odds of infections by MDROs were 19% and 41% higher in those with eGFR values between 30–60 ml/min/1.73 m^2^ (Adjusted odds ratio, AOR): 1.19, 95% CI:1.02–1.38, P = 0.022) and eGFR < 30 ml/min/1.73 m^2^ (AOR: 1.41, 95% CI:1.12–1.78, P = 0.004), respectively.

**Figure 3:**
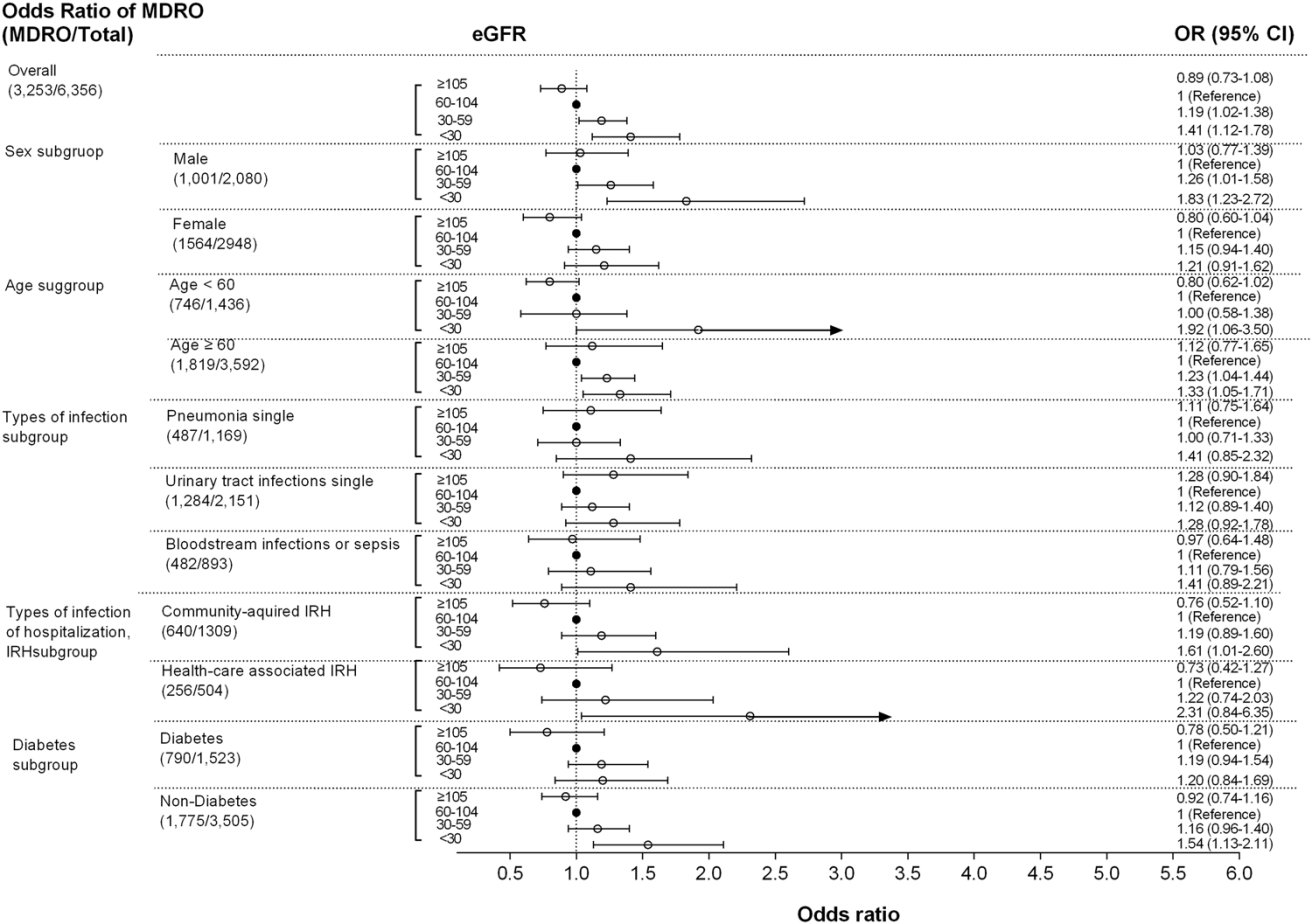
The odds ratio of MDROs in the first positive culture by different eGFR categories in Guangzhou, China. Sex subgroup adjusted by age and Charlson comorbidity index; Age subgroup adjusted by sex and Adjusted by Charlson comorbidity index; The rest of subgroups adjusted by age, sex and Charlson comorbidity index. *MDROs: multi-drug resistance organism; Defined by the following bacteria: *Staphylococcus aureus, Enterococcus spp., Enterobacteriaceae, Pseudomonas aeruginos, Acinetobacter spp*.; Resistance to three or more antimicrobial classes; IRH: infection-related hospitalization.

In subgroup analysis, the trends persisted across sex, age strata, single pneumonia, single urinary tract infections, sepsis, community-acquired infection, health-care associated infection and in patients with or without diabetes, although they did not reach statistical significance (Fig. 3).

In those with eGFR values determined 1–12 months prior to hospitalization, a sensitivity analysis demonstrated consistently higher risk of MDROs in those with lower eGFR categories, although the results were not statistically significant in this relatively small group (Supplementary Fig. 3). The higher odds of MDROs in those with low eGFR values at admission were observed, regardless of whether or not reduced renal function existed at a prior outpatient visit (Supplementary Fig. 4).

## Discussion

Our study shows that poorer kidney function at the time of hospital admission is associated with a higher probability of having MDROs. To our knowledge, this is the first study examining the association of kidney function at admission with infection by MDROs in adults without end-stage renal disease (ESRD). The finding in the current study of such an association is of potential clinical relevance. The main clinical application of this study lies in the need to consider the presence of renal dysfunction at admission as a signal to pay closer attention to the microbial culture results, given that identified pathogens may be potentially resistant to first-line initial antibiotics. Therefore, a subsequent modification of the initial antimicrobial therapy is needed if resistance to the current therapy exists. Our study also identifies patients with impaired renal function as a high-risk group for MDROs infections in need of close monitoring.

Previous studies have mainly focused on the higher risk of infection caused by MDROs, such as MRSA and VRE, in patients undergoing hemodialysis (1, 2)^11–14^. To our knowledge, there are no data to suggest whether or not the association may extend to milder levels of reduced renal function not requiring dialysis. Shorr *et al*. documented that long-term hemodialysis was an independent risk factor associated with infection due to antibiotic resistant bacteria^16^. An analysis from the CDC Active Bacterial Core surveillance (ABCs) system showed that the incidence of invasive MRSA infection in dialysis patients was 23.3 cases per 1000 persons, a rate that was 100 times greater than that in the general population^14^. Similar statistics have been reported from the United Kingdom (UK), where 4.2% of all episodes of MRSA bacteremia occurred among dialysis patients^17^. Our findings showed that higher odds of infection caused by MDROs were observed as the eGFR declined below 60 ml/min/1.73 m^2^, and were even higher in the group eGFR < 30 ml/min/1.73 m^2^ compared to the group with eGFR between 60–104 ml/min/1.73 m^2^.

Many factors might contribute to the higher odds of MDRO infections in patients with reduced renal function. Frequent exposure to antibiotics is the main driver of ABR^1^. Patients with reduced eGFR have a higher risk of antimicrobial exposure due to their susceptibility to infections^6,8,9,18–20^, either prescribed by physicians or self-prescribed. Infection susceptibility due to immune dysfunction has also been proposed in patients with impaired renal function, including increased production of reactive oxygen species (ROS), accumulation of toxic products, and functional abnormalities of monocytes, T lymphocytes and natural killer cells^21,22^. Furthermore, patients with more severely reduced renal function generally have more frequent contact with healthcare personnel and environments (e.g. hospitals), as well as other patients, due to comorbidities and complications^6^. This increases their potential exposure to MDROs. The high proportion of MDROs in our study is comparable to that of other studies in China (such as MDR *Acinetobacter baumannii:* 57.5–72.8%, MRSA: 44.6–73.5%)^23,24^. China is one of the leading antibiotic prescribing countries in the world and is facing the problem of misuse and overuse of antibiotics^25–27^. This might be the reason for the higher proportion of MDROs in the microbial results in our study than those in other western countries. The Chinese central government has started to address this problem with a more stringent antimicrobial stewardship policies since 2012, including restriction of antibiotic prescription to doctors, prevention of self-prescription, development of audit and inspection systems, and investigation and reassignment of responsibility to hospital management staff who violate rational use policies. These initiatives may take some time to mitigate the increased susceptibility of patients with reduced eGFR due to MDRO infection^24,28^. For physicians in clinical practice, close attention to eGFR at admission should inform clinical decision-making regarding rational prescription of initial and subsequent antibiotics in order to control the antibiotic resistance problem.

The strengths of this real-world study are its careful design and richness of information including microbial cultures from linked electronic medical databases. Although a high presence of MDROs has been documented in China, we were able to identify different odds or risks of MDROs across different eGFR strata even in the high MDRO context. Our findings are from Guangzhou, a city in the southern part of China, and may not be generalizable to other populations. However, we speculate that comparable associations may also be present in other populations with similar social status, income level and accessibility to antibiotics. The results should be interpreted considering the following limitations. (1) To define renal function, we used a single measurement of eGFR at admission, which may have resulted in misclassification of true eGFR as patients’ renal function may not have been in steady state at the time. Thus, it was not possible to determine with certainty whether patients with low eGFR values at hospital admission had acute kidney injury, chronic kidney disease or a combination of the two. A sensitivity analysis examining the relationship between eGFR values determined 1–12 months prior to hospitalization and MDROs demonstrated consistent findings to those of the main analysis, although the results were not statistically significant, possibly due to the more limited sample size. (2) We only focused on the first positive culture as the outcome, which was influenced by culture positive rate, previous hospitalization, antibiotic use before admission or admission from a nursing home^29–31^. There was no significant difference in culture positive rates among patients with different eGFR categories in our sample (Supplementary Table 3). However, we did not have access to a regional registry that allowed us to identify previous hospitalization and antibiotic use. (3) A positive culture could be related to contamination or colonization, rather than indicating the causative pathogen of infection. The possibility of a false positive culture result was mitigated by specifically matching a symptomatic clinical diagnosis with relevant types of positive culture samples. Residual and unknown confounding may have influenced our estimates. (4) As this is an observational study, the observed association between impaired renal function and MDROs cannot be used to draw causal inferences.

Our study suggests that patients with CKD at admission have a higher likelihood of having MDRO infections. This information may increase the awareness among physicians about the prognostic value of creatinine tests at admission, and aid in the choice of initial antibiotics.

## Methods

### Study Design

This was an observational study using electronic health records.

### Setting and data source

We used electronic healthcare data from four hospitals belonging to a healthcare conglomerate, Guangdong Provincial Hospital of Chinese Medicine (GDHCM), China. GDHCM is located in Guangzhou city with a population of 13,080,500 residents as of 2015^32^. GDHCM serves as one of the main referral centers for the area, with over 5 million outpatient visits and 70,000 inpatients per year. The four hospitals share the same electronic medical record database (EMRD), developed by International Business Machines Corporation (IBM), and which includes both inpatient and outpatient medical records.

### Participants

Patients were eligible for inclusion in the study if they were adults (≥18 years), were hospitalized with at least one discharge diagnosis of infection according to the *International Classification of Diseases, Tenth Revision, Clinical Modification* (ICD-10-CM), between August 2012 and December 2015 (see Supplementary Table 1 for definitions^33^), had a positive microbial culture result in which the type of sample matched the type of infection, and had at least one serum creatinine (sCr) measurement available at admission. Patients receiving renal replacement therapy (RRT, kidney transplantation or dialysis) were excluded. For patients with multiple hospital admissions, only the first admission record was used. The reasons why discharge diagnosis was used to confirm a hospitalization with infection included (1) need to capture all infections, including community acquired infection and health-care associated infection; (2) evidence was not always present to establish an infection diagnosis at admission. In the case of multiple infection diagnoses during the same hospitalization, only one infection-related diagnosis was used and only if the type of first positive culture aligned with the discharge diagnosis (Supplementary Table 2). For example, the first positive midstream urine culture should match to urinary tract infections; Bacteraemia/sepsis was valid if it had positive culture of any type and no other organ specific infection diagnosis. Thus, each hospitalization had a unique culture-confirmed infection diagnosis.

The protocol was reviewed and approved by the Ethical Committee of Guangdong Provincial Hospital of Chinese Medicine, China (B2016-194-01). Due to the retrospective nature of the study, informed consent was waived. All patient data were anonymized and de-identified prior to analysis.

### Renal function estimation

Estimated glomerular filtration rate (eGFR) was estimated from serum creatinine (sCr) concentration at admission (the first value was considered if more than one test was taken on the same day), using the CKD Epidemiology Collaboration (CKD-EPI) formula^34^. Patients were divided into four categories of eGFR: eGFR ≥ 105, 60–104, 30–59, and <30 ml/min/1.73 m^2^, with eGFR of 60–104 ml/min/1.73 m^2^ serving as the reference group because this range showed the lowest risk of infection in a previous study and higher eGFR, for example eGFR ≥ 105 ml/min/1.73 m^2^ might indicate malnutrition, which would be predisposed to high risk of infection^9^.

### Microbial susceptibility test and MDROs

Sampling and culture were performed according to standard procedures^35^. Microbiological tests based on Gram staining, microscopic observation of bacterial morphotypes, characteristics in culture medium and various specific biochemical reactions were applied to identify bacteria according to Clinical and Laboratory Standards Institute (CLSI) procedures^36^. Antibiotic susceptibility testing was also performed according to CLSI procedures, and micro-organisms were tested using a susceptibility panel^35^. The antibiotics panel used for susceptibility testing was applied according to CLSI guidelines and local prescription pattern. Minimum inhibitory concentrations (MICs) were determined by susceptibility analysis system (MicroScan® WalkAway® 96 Plus; Beckman Coulter, Brea, CA). The antibiotic susceptibility was interpreted using the CLSI MIC breakpoints at the time the test was conducted^35^. The standard and quality of the laboratory in GDHCM is certified by the International Organization for Standardization, ISO 15189.

MDROs were limited to a first culture positive pathogen of *Staphylococcus aureu*s, *Enterococcus spp*., *Enterobacteriaceae* (other than Salmonella and Shigella), *Pseudomonas aeruginosa* and *Acinetobacter spp*., and acquired non-susceptibility to at least one agent in three or more antibiotic classes, defined by European Centre for Disease Prevention and Control (ECDC) and the Centers for Disease Control and Prevention (CDC)^37^. The first positive culture was used to avoid the influence of the initial antimicrobial therapy on culture result and different culture results during the same hospitalization.

### Covariates

Patient age and sex were extracted from the medical records. According to the classification of Charlson comorbidities index using an established ICD-10 algorithm^38^, comorbid conditions were ascertained from the discharge diagnosis, including myocardial infarction, congestive heart failure, peripheral vascular disease, cerebrovascular disease, dementia, chronic pulmonary disease, rheumatologic disease, peptic ulcer disease, mild liver disease, diabetes without chronic complication, diabetes with chronic complication, hemiplegia or paraplegia, any malignancy, moderate or severe liver disease, metastatic solid tumor and AIDS/HIV. According to the GDHCM policy, discharge diagnosis was forced to include all the comorbid conditions from patients’ medical histories. Numbers of cultures, types of specimens and types of infection-related hospitalizations (IRHs) were extracted from the EMRD.

IRHs were sub-classified as health care-associated IRH (HAIRHs), community-acquired IRH (CAIRHs), and undefined IRH (UIRHs). HAIRHs were defined as the onset of infection after 48 hours following admission and confirmed by health care-associated infection report in the database which was audited by infection control healthcare professionals in GDHCM. CAIRHs were defined as those with infection diagnosis at admission. UIRHs were those that did not fulfill any of the criteria above.

### Statistical analysis

Numerical variables were summarized using mean ± standard deviation, or median and interquartile range, as appropriate, while categorical variables were summarized using proportions. Differences in baseline characteristics and the proportion of MDROs in the first culture result between groups of patients within the 4 different eGFR categories were compared using ANOVA, Mann-Whitney U test, chi-square test, Fisher´s exact test, or Wilcoxon rank-sum test, as appropriate. All the data extracted from EMRD in our study were complete, with no missing data and loss to follow-up.

Multivariable logistic regression was used to calculate the adjusted odds ratio (AOR) of MDROs in the first culture result among different categories of eGFR. Covariates included age group (18–44; 45–59; 60–74; ≥75 years), sex, and Charlson comorbidity index (excluding the renal disease score). A P-value < 0.05 was considered significant. Additionally, we repeated the main analysis in subgroups: different sex, age groups, diabetes groups, cause-specific infection-related outcomes including blood stream infections or sepsis, pneumonia and urinary tract infections and types of hospitalization. In light of the fact that reduced eGFR values at admission may have represented pre-existing renal impairment, we performed a sensitivity analysis in those with and without pre-existing reduced renal function. Reduced renal function at admission without pre-existing renal impairment was defined as eGFR 60–104 ml/min/1.73 m^2^ at outpatient visit 1–12 months preceding hospitalization and eGFR < 60 ml/min/1.73 m^2^ at admission. Reduced renal function at admission with pre-existing reduced renal function was defined as eGFR < 60 ml/min/1.73 m^2^ both at preceding outpatient visits and at admission. We also performed a sensitivity analysis to estimate the relative risk of MDROs in the first culture result across different eGFR strata using log binomial model. All statistical analyses were performed using STATA version 14.2 (StataCorp, College Station, TX, USA).

## Electronic supplementary material

Supplementary materials

## Data Availability

The datasets used and/or analysed during the current study are available from the corresponding author on reasonable request and with permission of Guangdong provincial hospital of Chinese medicine.
