# Supplementary material for: Association of Kidney Function with Infections by Multidrug-Resistant Organisms: An Electronic Medical Record Analysis
Source: Sci Rep. 2018 Sep 6;8:13372. doi: 10.1038/s41598-018-31612-1 (PMC6127257; doi:10.1038/s41598-018-31612-1)
Supplement: Supplementary file 1 — Supplementary materials [file 41598_2018_31612_MOESM1_ESM.pdf]

**Association of Kidney Function with Infections by Multidrug-Resistant Organisms: An Electronic Medical Record Analysis**

Guobin Su[1,2] MD, Hong Xu[3,4] MD, Emilia Riggi[1,5], Zhiren He[2] MD, Liming Lu[6] PhD, Prof Bengt Lindholm[4] MD PhD, Gaetano Marrone[1] PhD, Prof Zehuai Wen[6] PhD, Prof Xusheng Liu[2] MD, Prof David W Johnson[7,8,9] MD PhD, Juan-Jesus Carrero[3,4] PhD, Prof Cecilia Stålsby Lundborg[1] PhD

[1] Global Health – Health Systems and Policy, Department of Public Health Sciences, Karolinska Institutet, Stockholm, Sweden;

[2] Department of Nephrology, Guangdong Provincial Hospital of Chinese Medicine, The Second Affiliated Hospital, Guangzhou University of Chinese Medicine, Guangzhou city, Guangdong Province, China;

[3] Department of Medical Epidemiology and Biostatistics, Karolinska Institutet, Stockolm, Sweden;

[4] Division of Renal Medicine and Baxter Novum, Department of Clinical Science, Intervention and Technology, Karolinska Institutet, Stockholm, Sweden;

[5]Department of Brain and Behavioral Sciences, Unit of Medical Statics and Genomics, University of Pavia, Italy

[6] Key Unit of Methodology in Clinical Research (KUMCR), Guangdong Provincial Hospital of Chinese Medicine, The Second Affiliated Hospital, Guangzhou University of Chinese Medicine, Guangzhou city, Guangdong Province, China.

[7] Department of Nephrology, Princess Alexandra Hospital, Brisbane, Australia

[8] Centre for Kidney Disease Research, University of Queensland, Brisbane, Australia

[9] Translational Research Institute, Brisbane, Australia

**Corresponding author:**

Prof Xusheng Liu

Department of Nephrology, Guangdong Provincial Hospital of Chinese Medicine, The Second Affiliated Hospital, Guangzhou University of Chinese Medicine, Guangzhou city, Guangdong Province, China;

Tel:+86-81887233-34330 Fax: +86-81887233-34330

Email: xushengliu801@126.com or liuxusheng@gzucm.edu.cn

**Supplementary Table 1. ICD-10 codes for all and different types of infections.**

We limited our examination to the infection-related diagnoses outlined. It has been used previously. The following discharge diagnoses were not considered in our study: pregnancy-related infections, delivery-related infections, oral/mouth infections, ear infections, eye infections, pancreatitis, thyroiditis, pituitary gland, infections specified as chronic, chronic hepatitis B and C virus, HIV (human immunodeficiency virus), cholecystitis associated with cholelithiasis/choledocholithiasis, sexually transmitted infections, parasitic or protozoal diseases and device-dialysis related infection.

| Infection categories | ICD-10 codes |
|---|---|
| Respiratory tract infections | |
| | |
| Non-pneumonia respiratory tract infections | A06.5 A20.2 A21.2 A36.0 A36.1 A36.2 A36.8 A36.9 A37.0 A37.1 A37.8 A37.9 A38.x B00.2 B08.5 B33.4 B44.2 B48.1 J10.8 J00.x J01.0 J01.1 J01.2 J01.3 J01.4 J01.8 J01.9 J02.0 J02.8 J02.9 J03.0 J03.8 J03.9 J04.0 J04.1 J04.2 J05.0 J05.1 J06.0 J06.8 J06.9 J09.x J10.1 J11.1 J11.8 J20.0 J20.1 J20.2 J20.3 J20.4 J20.5 J20.6 J20.7 J20.8 J20.9 J21.0 J21.8 J21.9 J22.x J34.0 J39.0 J39.1 J40.x J41.1 J44.0 J47.x J85.2 J85.3 J86.0 J86.9 J38.3 J38.7 J39.2 J39.8 J95.0 J98.5 J98.9 |
| Pneumonia | A22.1 A31.0 A42.0 A43.0 A48.1 B01.2 B05.2 B25.0 B37.1 B38.0 B38.2 B39.2 B40.2 B41.0 B42.0 B44.0 B44.1 B45.0 B46.0 J10.0 J11.0 J12.0 J12.1 J12.2 J12.8 J12.9 J13.x J14.x J15.0 J15.1 J15.2 J15.3 J15.4 J15.5 J15.6 J15.7 J15.8 J15.9 J16.0 J16.8 J18.0 J18.1 J18.2 J18.8 J18.9 J85.0 J85.1 J98.4 |
| Genitourinary infections | |
| Urinary tract infections (UTIs) | N10.x N12.x N13.6 N15.1 N30.0 N30.3 N34.0 N34.1 N34.2 N34.3 N39.0 N15.9 N28.8 N30.8 N30.9 |
| Non-UTIs genitourinary infections | B26.0 B37.3 B37.4 N41.0 N41.2 N41.3 N41.8 N41.9 N43.1 N45.0 N45.9 N48.1 N48.2 N49.0 N49.1 N49.2 N49.8 N49.9 N61.x N70.0 N70.9 N71.0 N71.9 N72.x N73.0 N73.2 N73.3 N73.5 N73.8 N73.9 N75.1 N76.0 N76.2 N76.4 N48.0 N75.8 N76.8 N32.3 |
| Bloodstream infections or sepsis; | A02.1 A20.7 A21.7 A22.7 A26.7 A32.7 A39.1 A39.2 A39.4 A40.0 A40.1 A40.2 A40.3 A40.8 A40.9 A41.0 A41.1 A41.2 A41.3 A41.4 A41.5 A41.8 A41.9 A42.7 A48.3 B00.7 B37.7 R65.1 R65.0 R65.2 U04.9 |
| Abdominal infections | A00.0 A00.1 A00.9 A01.0 A01.1 A01.2 A01.3 A01.4 A02.0 A03.0 A03.1 A03.2 A03.3 A03.8 A03.9 A04.0 A04.1 A04.2 A04.3 A04.4 A04.5 A04.6 A04.7 A04.8 A04.9 A05.0 A05.1 A05.2 A05.3 A05.4 A05.8 A05.9 A06.0 A06.2 A06.3 A06.4 A07.0 A07.1 A07.2 A07.3 A07.8 A07.9 A08.0 A08.1 A08.2 A08.3 A08.4 A08.5 A09.0 A09.9 A21.3 A22.2 A42.1 B05.4 B15.0 B15.9 B16.0 B16.1 B16.2 B16.9 B17.0 B17.1 B17.2 B17.8 B17.9 B19.0 B19.9 B25.1 B25.8 B46.2 K35.0 K35.1 K35.9 K36.x K37.x K57.0 K57.2 K57.4 K57.8 K61.0 K61.1 K61.2 K61.3 K61.4 K63.0 K65.0 K65.9 K75.0 K81.0 K81.8 K81.9 K52.1 K52.9 K57.1 K57.3 K62.8 K63.8 K65.8 K83.0 |
| Skin and soft tissue infections | A06.7 A20.1 A22.0 A26.0 A26.8 A26.9 A31.1 A32.0 A36.3 A43.1 A44.1 A46.x A48.0 B00.0 B00.1 B02.9 B07.x B08.8 B09.x B35.0 B35.1 B35.2 B35.3 B35.4 B35.5 B35.6 B35.8 B35.9 B36.0 B36.1 B36.2 B36.3 B36.8 B36.9 B37.2 B38.3 B40.3 B42.1 B43.0 B43.2 B45.2 B46.3 B48.0 L00.x L01.0 L01.1 L02.0 L02.1 L02.2 L02.3 L02.4 L02.8 L02.9 L03.0 L03.1 L03.2 L03.3 L03.8 L03.9 L04.0 L04.1 L04.2 L04.3 L04.8 L04.9 L05.0 L05.9 L08.0 L08.1 L08.8 L08.9 L30.3 L70.2 R02.x L40.1 L40.3 L70.0 L73.2 L84.x L88.x L98.0 |
| Nervous system infections; | A06.6 A20.3 A32.1 A39.0 A83 A85.8 A86.x A87.0 A87.1 A87.2 A87.8 A87.9 A88.8 A89.x B00.3 B00.4 B01.0 B01.1 B02.0 B02.1 B02.2 B05.0 B05.1 B06.0 B26.1 B26.2 B37.5 B38.4 B43.1 B45.1 B46.1 G00.0 G00.1 G00.2 G00.3 G00.8 G00.9 G04.0 G04.2 G06.0 G06.1 G06.2 G93.7 F05.9 G03.9 G04.8 G04.9 G62.9 |
| Musculoskeletal infections | B33.0 M00.0 M00.1 M00.2 M00.8 M00.9 M46.2 M46.3 M46.5 M60.0 M65.0 M65.1 M71.0 M71.1 M86.0 M86.1 M86.2 M86.8 M86.9 M46.8 M72.8 M94.8 |
| Cardiovascular infections | A39.5 B33.2 B37.6 I01.0 I01.1 I01.2 I01.8 I01.9 I02.0 I02.9 I30.1 I33.0 M05.3 M32.1 I40.0 I410, I411, I412, I430, I39 |
| Other infections of interest | A02.2 A02.8 A02.9 A06.8 A06.9 A20.0 A20.8 A20.9 A21.0 A21.8 A21.9 A22.8 A22.9 A23.0 A23.1 A23.2 A23.3 A23.8 A23.9 A24.0 A24.1 A24.3 A24.4 A25.0 A25.1 A25.9 A27.0 A27.8 A27.9 A28.0 A28.1 A28.2 A28.8 A28.9 A30.0 A30.1 A30.2 A30.3 A30.4 A30.5 A30.8 A30.9 A31.8 A31.9 A32.8 A32.9 A35.x A39.8 A39.9 A42.2 A42.8 A42.9 A43.8 A43.9 A44.0 A44.8 A44.9 A48.2 A48.4 A48.8 A49.0 A49.1 A49.2 A49.3 A49.8 A49.9 A88.0 A88.1 A90.x A91.x A92.0 A92.1 A92.2 A92.3 A92.4 A93.0 A93.1 A93.2 A93.8 A95.0 A95.1 A95.9 A96.0 A96.1 A96.2 A96.8 A96.9 A98.0 A98.1 A98.2 A98.3 A98.4 A98.5 A98.8 A99.x B00.8 B00.9 B01.8 B01.9 B02.7 B02.8 B03.x B04.x B05.8 B05.9 B06.8 B06.9 B08.0 B08.1 B08.2 B08.3 B08.4 B25.9 B26.8 B26.9 B27.0 B27.1 B27.8 B27.9 B33.3 B33.8 B34.0 B34.1 B34.2 B34.3 B34.4 B34.8 B34.9 B37.0 B37.8 B37.9 B38.7 B38.8 B38.9 B39.0 B39.3 B39.4 B39.5 B39.9 B40.0 B40.7 B40.8 B40.9 B41.7 B41.8 B41.9 B42.7 B42.8 B42.9 B43.8 B43.9 B44.7 B44.8 B44.9 B45.3 B45.7 B45.8 B45.9 B46.4 B46.5 B46.8 B46.9 B47.0 B47.1 B47.9 B48.2 B48.3 B48.4 B48.7 B48.8 B49.x B95.0 B95.1 B95.2 B95.3 B95.4 B95.5 B95.6 B95.7 B95.8 B96.0 B96.1 B96.2 B96.3 B96.4 B96.5 B96.6 B96.7 B96.8 B97.0 B97.1 B97.2 B97.3 B97.4 B97.5 B97.6 B97.7 B97.8 B99.x D73.3 E32.1 I00.x I83.2 K11.2 R17.x D76.2 |

**Supplementary Table 2. The link between infection diagnosis and culture sample.**

| Discharge infection diagnosis | Type of culture sample |
|---|---|
| Respiratory tract infections | Sputum, Nasopharyngeal swab |
| Urinary tract infections (UTIs) | Midstream urine |
| Non UTIs genitourinary infections | Cervical swab, Vaginal swab |
| Bloodstream infections or sepsis; | All types of sample |
| Abdominal infections | Stool, Bile, Ascitic fluid |
| Skin and soft tissue infections | Wound swab, Skin and soft tissue |
| Nervous system infections; | Cerebrospinal fluid |
| Musculoskeletal infections | Joint fluid, muscle tissue |
| Cardiovascular infections | Pericardial effusion |

**Supplementary Table 3. Culture positive rate in the incident infection-related hospitalizations, among patients with different kidney function at admission.**

| eGFR at admission | Overall culture positive, n (%) | ≥105 (n=18,174) | 60-104 (n=52,642) | 30-59 (17,286) | < 30 (n=6,343) | P value |
|---|---|---|---|---|---|---|
| Total (n=94,445) | 20,547 (21.8) | 3,844 (21.2) | 11457 (21.8) | 3,805 (22.0) | 1,441 (22.7) | 0.06 |
| Sputum (n=44,412) | 9,226 (20.8) | 1,498 (19.8) | 5,422 (20.7) | 1,731 (21.2) | 575 (22.89) | ＜0.01 |
| Mid-stream urine (n=15,981) | 4,844 (30.3) | 718 (29.7) | 2,667 (30.8) | 987 (29.9) | 472 (29.8) | 0.60 |
| Venous blood (n=13,064) | 1,245 (10) | 234 (9.1) | 678 (9.8) | 235 (9.4) | 98 (9.5) | 0.70 |
| Other types of specimen (n=20,988) | 5,232 (25) | 1,394 (24.8) | 2,690 (24.8) | 852 (25.8) | 296 (24.5) | 0.70 |

**Supplementary Figure 1. Pattern of cultured acertained infection by different eGFR categories in Guangzhou, China.**

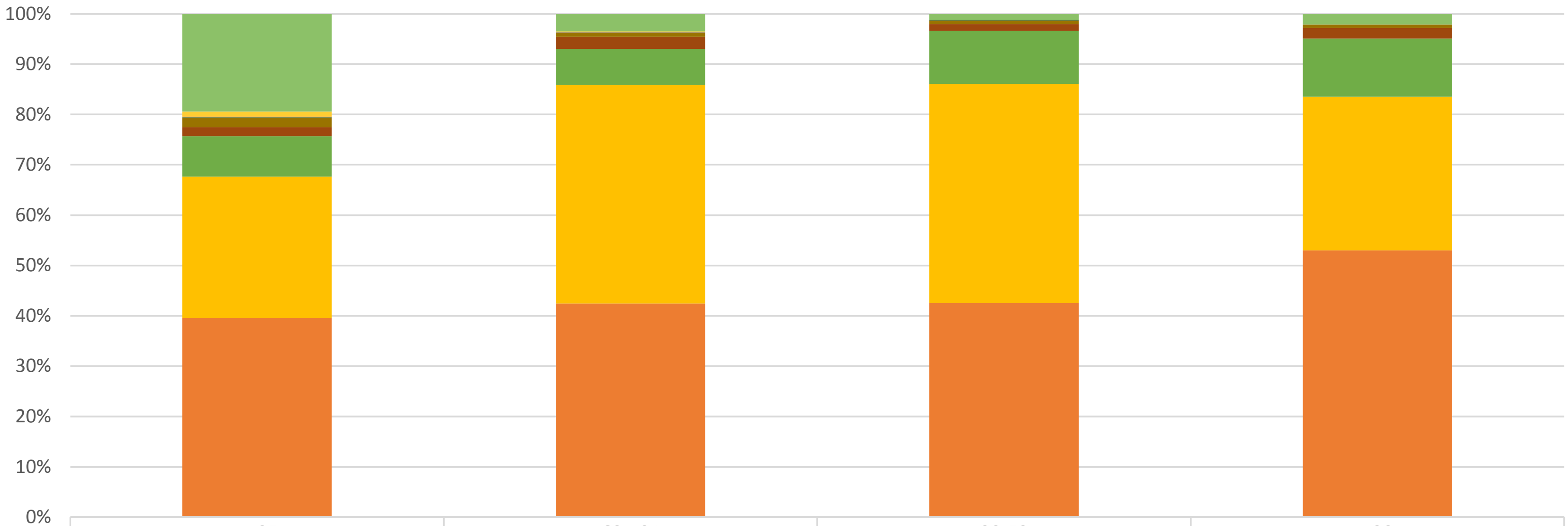


| | ≥105 (n=1,231) | 60-104 (n=4,207) | 30-59 (n=1,382) | <30 (n=468) |
|---|---|---|---|---|
| Other infections,% | 19.42 | 3.49 | 1.23 | 2.14 |
| Nervous system,% | 0.97 | 0.12 | 0 | 0 |
| Musculoskeletal,% | 0.16 | 0.1 | 0.07 | 0 |
| Cardiovascular,% | 0.08 | 0.07 | 0.14 | 0 |
| Skin and soft tissue,% | 1.95 | 0.76 | 0.65 | 0.64 |
| Abdominal,% | 1.71 | 2.4 | 1.3 | 2.14 |
| Bloodstream or sepsis,% | 8.04 | 7.23 | 10.56 | 11.54 |
| Respiratory tract,% | 28.11 | 43.36 | 43.56 | 30.56 |
| Genitourinary,% | 39.56 | 42.48 | 42.47 | 52.99 |

Genitourinary,% Respiratory tract,% Bloodstream or sepsis,% Abdominal,% Skin and soft tissue,%
Cardiovascular,% Musculoskeletal,% Nervous system,% Other infections,%

**Supplementary Figure 2. The first positive culture pattern in all samples by different eGFR categories in Guangzhou, China.**

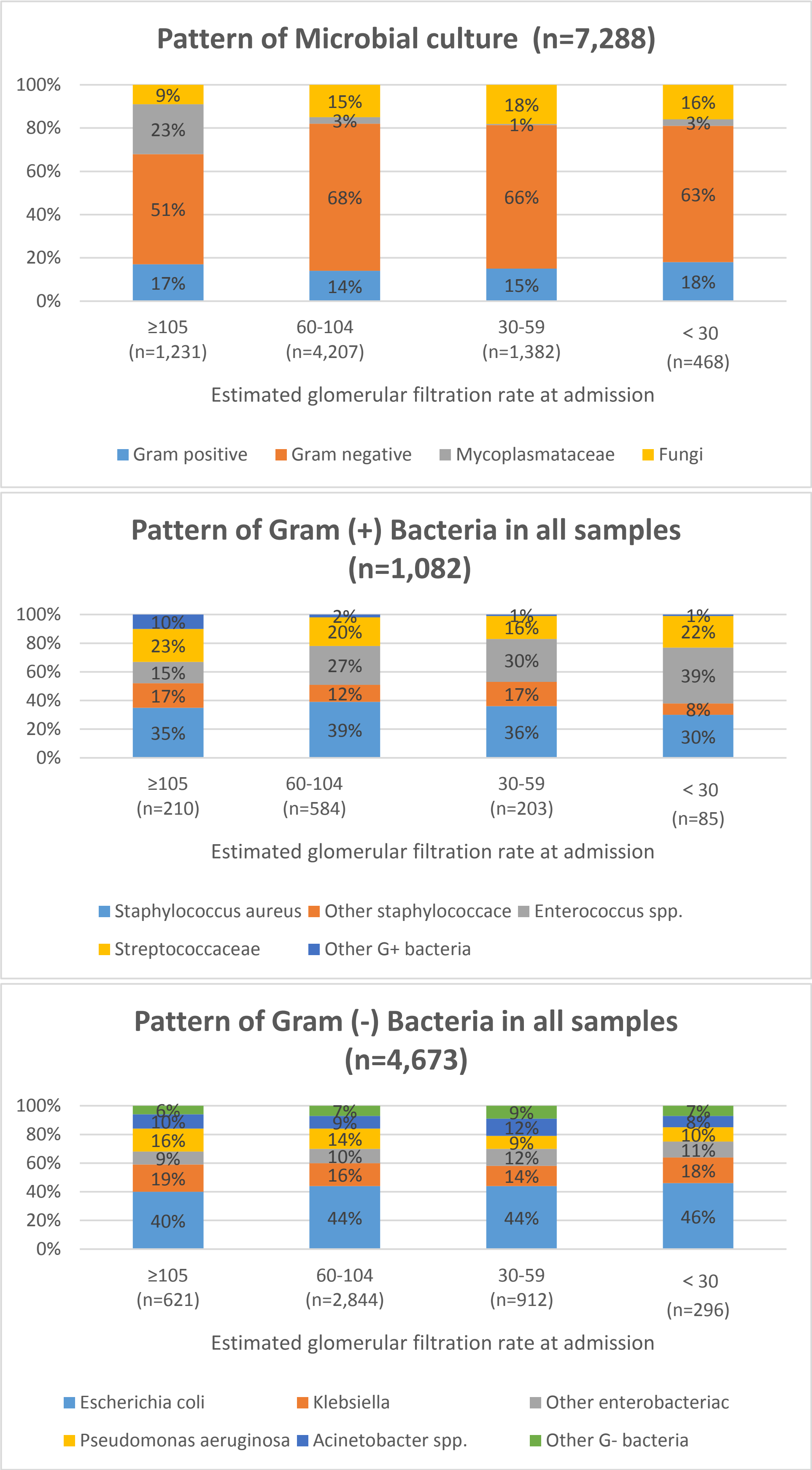

**Supplementary Figure 3. Relative risk of MDROs in the first positive culture by eGFR categories.**

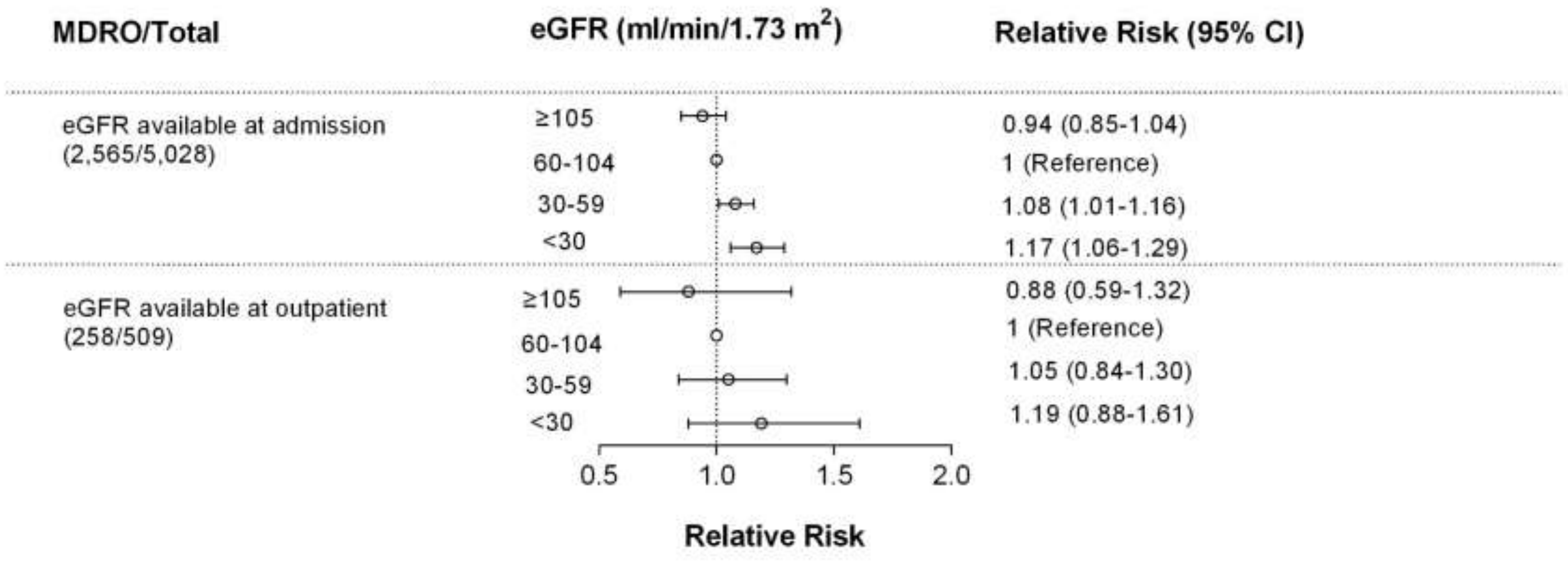


Log binomial model adjusted by Age, sex and Charlson comorbidity index

*MDROs: multi-drug resistance organism; Defined by the following bacteria: *Staphylococcus aureus, Enterococcus spp., Enterobacteriaceae, Pseudomonas aeruginos, Acinetobacter spp.*; Resistance to three or more antimicrobial classes;

**Supplementary Figure 4. Odds ratio of MDROs in the first positive culture by pre-existing reduced renal function at outpatients 1-12 months before hospitalization**

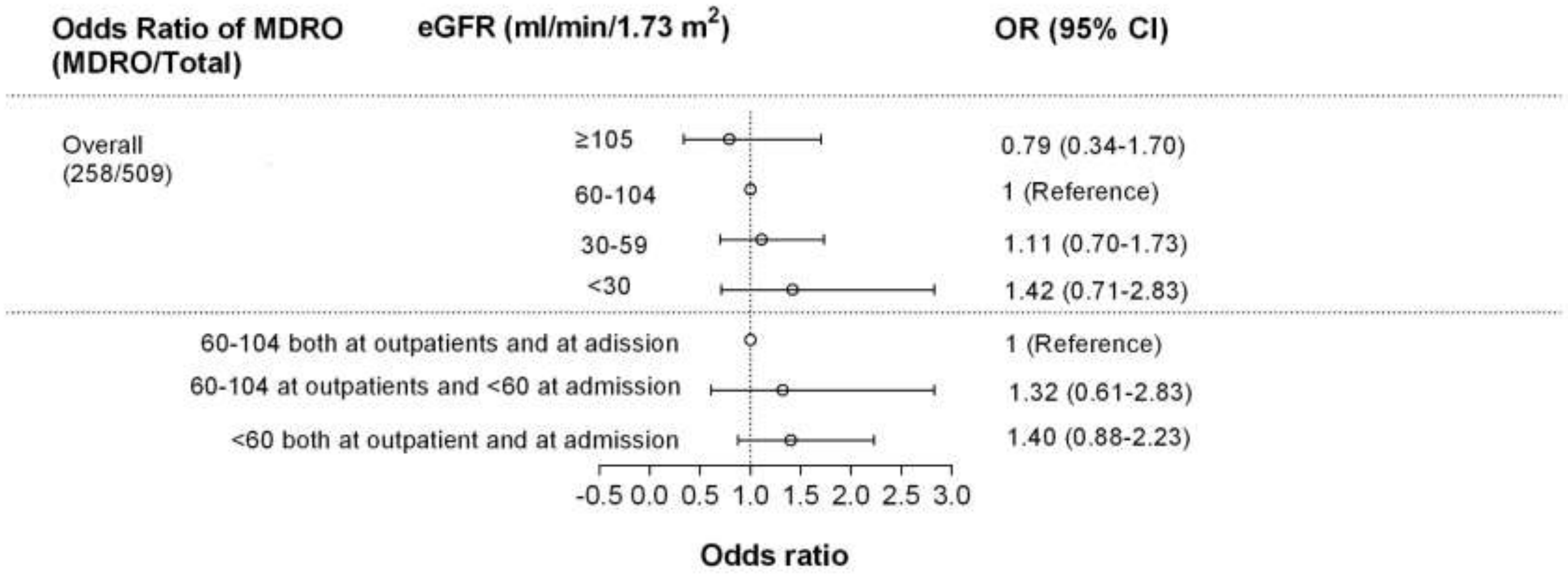


Multivariable logistic regression adjusted by Age, sex and Charlson comorbidity index
*MDROs: multi-drug resistance organism; Defined by the following bacteria: *Staphylococcus aureus, Enterococcus spp., Enterobacteriaceae, Pseudomonas aeruginosa, Acinetobacter spp.*; Resistance to three or more antimicrobial classes;
